# bioNMF: a versatile tool for non-negative matrix factorization in biology

**DOI:** 10.1186/1471-2105-7-366

**Published:** 2006-07-28

**Authors:** Alberto Pascual-Montano, Pedro Carmona-Saez, Monica Chagoyen, Francisco Tirado, Jose M Carazo, Roberto D Pascual-Marqui

**Affiliations:** 1 Computer Architecture Department, Facultad de Ciencias Físicas, Universidad Complutense de Madrid, 28040, Spain; 2 BioComputing Unit, National Center of Biotechnology, Campus Universidad Autónoma de Madrid, 28049, Spain; 3 The KEY Institute for Brain-Mind Research, University Hospital of Psychiatry. Lenggstr. 31, CH-8029 Zurich, Switzerland

## Abstract

**Background:**

In the Bioinformatics field, a great deal of interest has been given to Non-negative matrix factorization technique (NMF), due to its capability of providing new insights and relevant information about the complex latent relationships in experimental data sets. This method, and some of its variants, has been successfully applied to gene expression, sequence analysis, functional characterization of genes and text mining. Even if the interest on this technique by the bioinformatics community has been increased during the last few years, there are not many available simple standalone tools to specifically perform these types of data analysis in an integrated environment.

**Results:**

In this work we propose a versatile and user-friendly tool that implements the NMF methodology in different analysis contexts to support some of the most important reported applications of this new methodology. This includes clustering and biclustering gene expression data, protein sequence analysis, text mining of biomedical literature and sample classification using gene expression. The tool, which is named bioNMF, also contains a user-friendly graphical interface to explore results in an interactive manner and facilitate in this way the exploratory data analysis process.

**Conclusion:**

bioNMF is a standalone versatile application which does not require any special installation or libraries. It can be used for most of the multiple applications proposed in the bioinformatics field or to support new research using this method. This tool is publicly available at .

## Background

The development of "omics" technologies has represented a revolution in biomedical research allowing the study of biological systems from a global perspective. These high-throughput techniques generate vast amounts of data which have required the development and application of sophisticated statistical and machine learning methodologies in order to analyze and extract biological knowledge.

Matrix factorization techniques have become well established methods for the analysis of such datasets. These methods can be applied to the analysis of multidimensional datasets in order to reduce the dimensionality, discover patterns and aid in the interpretation of the data. Among the most popular, Principal Component Analysis (PCA), Singular Value Decomposition (SVD) or Independent Component Analysis (ICA) have been successfully used in a broad range of contexts such as transcriptomics [1-3], metabolomics [4,5] or proteomics [6].

In 1999 Lee and Seung developed a novel matrix factorization technique named Non-Negative Matrix Factorization (NMF) [7] to decompose images into recognizable features. The main difference between NMF and other classical factorization methods relies on the non-negativity constraints imposed to the model. These constraints tend to lead to a parts-based representation of the data because they allow only additive, not subtractive, combinations of data items. In this way, the factors produced by this method can be interpreted as parts of the data or, in other words, as subsets of elements that tend to occur together in sub-portions of the dataset (see implementation section for details). On the contrary, classical factorization techniques decompose the data matrix into a new set of matrices of any sign, involving complex cancellations of positive and negative elements to reconstruct the original dataset. Therefore the interpretation of the factors becomes non-intuitive and difficult [7,8]. The comprehensible properties of the NMF method and the intuitivism of the results it provides have centered the attention of many researches in different fields of science and, in particular, in the bioinformatics field where NMF has been applied to the analysis of gene expression data [9-15], protein sequences [16], functional categorization of genes [17] or text mining [18].

Despite the increasing use of NMF in Bioinformatics, most of its implementations are only available as MATLAB (Mathworks, Natick, MA) toolboxes, command line programs [12] or integrated in larger analysis packages [19]. Even if these solutions are useful, there are situations in which a simple standalone tool to perform a very specific type of analysis, as the ones described in the literature [9-18], is still needed. This situation has motivated us to develop bioNMF, a user-friendly tool that implements the classical NMF algorithm and a new sparse variant, the Non-Smooth Non-Negative Matrix factorization [20] tailored for different applications proposed in the bioinformatics field.

## Implementation

bioNMF has been implemented as a single standalone application for Microsoft Windows platform. The application has been written in Borland Delphi version 7 and it does not require any special installation or libraries and thus bioNMF is self-contained in a single application file. Analysis using bioNMF can be executed in three steps:

1) *Selection of the data set for analysis*: bioNMF accepts as input data files tab separated text files, which might or might not contain row and column labels. In addition and for compatibility reasons, it also accepts data in the format used by the Eng*e*ne software package [21], which allows multiple annotations per rows and columns. There is no limit, in principle, on the size of the input data (number of rows and columns). Only available memory might practically limits its size.

2) *Transformation of the data for normalization and to accommodate it for positive constraints*: Seven normalization methods have been included to pre-process the data before the analysis. After normalization, data can be accommodated to satisfy the non-negative constraints necessary for the NMF algorithm.

3) *Run analysis*: the analysis step has been divided into three main modules, which comprises most of the most important NMF applications proposed so far [9,10,13,16-18]: a) Standard NMF; b) Biclustering Analysis; c) Sample classification. All of them make use of the Non-negative matrix factorization model described below.

### Non negative matrix factorization model

NMF is a matrix factorization algorithm originally introduced in [7]. This technique can be applied to the analysis of multidimensional datasets in order to reduce the dimensionality, discover latent patterns and, more important, aid in the interpretation of the data. Formally, the non-negative matrix decomposition can be described as: **V ≈ WH **where **V **∈ ℝ^*m*×*n *^is a positive data matrix with *m *variables and *n *objects, **W **∈ ℝ^*m*×*k *^are the reduced *k *basis vectors or factors, and **H **∈ ℝ^*k*×*n *^contains the coefficients of the linear combinations of the basis vectors needed to reconstruct the original data (also known as encoding vectors). The main difference between NMF and other classical factorization models relies in the non-negativity constraints imposed on both the basis **W **and encoding vectors **H**. In this way, only additive combinations are possible. The number of factors (*k*) is generally chosen so that it takes a value less than *n *and *m*. This is usually imposed since the one of the main purpose of this model is to reduce the overall dimensionality of the data.

In the case of gene expression analysis, for example, the expression data matrix **V **can be represented as a gene-experiment matrix, where *m *is the number of genes and *n *the number of experimental conditions. The *k *columns of **W**, therefore, will have the dimension of a single array (*m *genes) and are known as basis experiments or factors. Similarly, the *n *columns of **H **are known as encoding vectors and are in one-to-one correspondence with a single experiment of the gene expression data matrix. Consequently, each row of **H **has the dimension of a single gene (*n *experiments) and it is denoted as basis gene.

## Results and discussion

**Figure 1:**
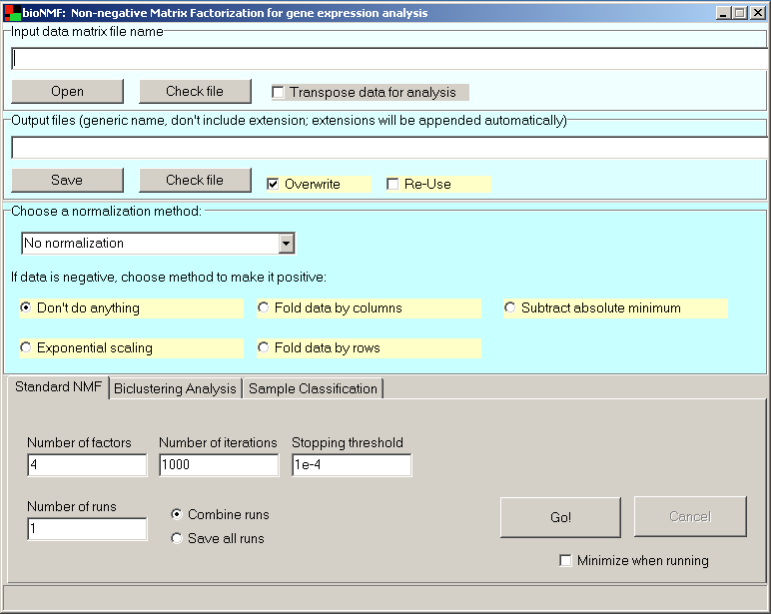
**Main bioNMF window**. The tool is divided into three main functional modules: data input, data transformation and analysis modules (standard NMF, bicluster analysis and sample classification). Standard NMF implements the classical Lee and Seung NMF algorithm. Bicluster analysis uses a sparse variant of the NMF model while Sample classification implements an unsupervised classification method that uses NMF to classify experimental samples.

The main window of bioNMF application is divided into three groups: data input, data transformation and analysis (see Figure 1). The application is able to read the data matrix in raw text format with or without header rows and columns. Data can also be transposed for analysis. Even if NMF seems to be an robust algorithm [9], specially if compared with other clustering methods like hierarchical clustering or SOM, a previous normalization step is usually necessary to make more evident the patterns of interest. Therefore data normalization is provided as a pre-processing step before computing the factorization. Normalization methods provided include data centering, standardization of rows and columns (independently or simultaneously), mean subtraction by rows and columns and the normalization method proposed in [22] that first divide each column by its mean and then normalize each row. After the normalization step, if the data contains negative values, this tool offers a set of methodologies to make it positive:

• *Subtract absolute minimum*: The minimum negative value is subtracted to every single cell of the data matrix (global additive shift) [19].

• *Fold data by rows*: This approach was used by Kim and Tidor for the analysis of log-transformed gene expression data [13]. Every row (items) is represented in two new rows of a new matrix. The first one is used to indicate positive expression (up-regulation) and the second one to indicate a negative expression value (down-regulation). This process doubles the number of rows of the data set.

• *Fold data by columns*: In a similar way, this option allows users to make the data positive by folding columns (variables). Every column is then represented in two new columns. The first occurrence to indicate positive expression and the second to indicate negative values, doubling in this way the number of columns of the data set.

• *Exponential scaling*: Data is exponentially scaled to make it positive. This is an inverse operation of a logarithmic transformation.

The following section describe in details the three main analysis modules implemented in bioNMF. More information and step by step examples are included in the project web site.

### Standard NMF

This module performs the classical NMF factorization using the algorithm proposed by Lee and Seung [7]. In the context of Bioinformatics most of the reported NMF applications were intended to create a new representation space in which to perform further analysis. Generally, this new space has several advantages compared to the initial representation (e.g. dimension reduction, latent semantics, part-based decomposition). Therefore, the exact nature of input data matrices, as well as post-processing of bioNMF output will depend on the particular application field, where other analysis tools can be used. To exemplify the type of analysis problems where the NMF algorithm has been successfully applied, we include a brief description of four applications in different contexts in biology: gene expression data analysis, biomedical text analysis, protein sequence analysis and functional characterization of genes.

Kim and Tidor applied NMF for gene expression data analysis in yeast [13]. They analyzed a data set containing expression patterns monitored for 6316 *Saccharomyces cerevisiae *genes in 300 experiments involving a variety of strains and conditions [23]. Expression values represented the ratio of the expression in the experiment to that in a control experiment of wild type grown under standard conditions. In this particular case, they used expression matrix **V **as an *n *× *m *matrix, where *m *corresponded to arrays (i.e., experiments) and *n *to genes. In this application they verified that NMF is able to recognize localized gene expression features that are dominated by a few functional categories, indicating that they represent a grouping of genetic components on the basis of cellular function [13]. The novelty of this approach with respect to other clustering methods relies on the fact that NMF produces clusters based on local patterns, grouping sets of genes that behave in a strongly correlated fashion in only sub-portions of the data. They showed that the prediction of functional relationships between experiments using the reduced space yielded by NMF outperforms some conventional approaches.

Another NMF application proposed in the context of data analysis in biology is text mining [18]. In this case, input data matrix **V **can be modeled as a gene-document collection represented in a vector space model, where **V **is an *m*×*n *sparse matrix (*m *representing the total number of terms in the literature corpus and *n *representing the genes). NMF has been used in this context to find relevant common sub-sets of terms that correspond to latent concepts in the literature corpus relevant to the genes.  Therefore, any vector space model, using certain weighting scheme, can be provided to bioNMF.

Given a factorization rank (*k*) selected by the user, *Standard NMF *analysis will produce two new matrices (**W**, **H**). Each factor (column) in the matrix **W **corresponds to a semantic feature (described as weighted sum of terms) while each column in **H **corresponds to the new representation for a gene as a linear combination of semantic factors (gene semantic profile). To provide both a more comprehensive representation of the genes, and a more robust clustering, Chagoyen *et al*. 2006 [18] constructed semantic profiles of gene-documents by combining the results from independent runs of the NMF algorithm, using the same number of factors at each run. For that reason, and to provide bioNMF with this capability, NMF can be executed a predetermined number of times with different random initial conditions and results can be saved separately or combined in a single file. In this way additional analysis, for example clustering can be performed using the combination of **H **matrices from different random runs. E.g. clustering of genes according to similar semantic profiles.

NMF analysis has also being used for the identification of sequence patterns conserved in subgroups of proteins in diverse superfamilies [16]. In this case, input data matrix **V **corresponds to a generalized sequence space (*m *attributes, *n *proteins) that can be obtained from a fuzzy alignment as defined by Heger and Holm, 2003 [16]. This fuzzy alignment model probabilistically assigns residues to structurally equivalent positions (attributes) of the proteins. Columns of **W **are the basis vectors of the reduced space, and **H **is the encoding in the new basis. The coefficients of the attributes in a basis vector (column in **W**) reflect the frequency of particular residues in the corresponding protein set. In [16], attention was focused on a small set of attributes, selected by covariance analysis of **W **matrix obtained at different ranks (namely *k *= 2, 4, 8, 16, 32 and 128). The resulting clusters of attributes represented conserved sequence patterns.

Finally, NMF has been described to perform functional categorization of genes [17]. Input matrix **V **of size *m*×*n *(*m *genes, *n *functional classes) corresponds to a binary matrix of functional associations of genes to their corresponding GO terms (in the three ontologies). **W **describes the loadings of the genes on the *k *factors and it is further used in cluster analysis. In the clustering process, the genes are deposited into clusters by using a winner-takes-all approach that finds the factor with the highest loading for each gene from matrix **W**, providing in this way insights about the most prominent functional categories for each gene.

**Figure 2:**
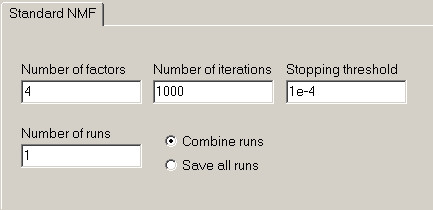
**Standard NMF module**. This functional module implements the classical NMF algorithm. Different random runs can be executed and results can be either combined in a single output file or saved independently. The application selects the best run based on the minimum error of the model.

Due to the non-deterministic nature of NMF results might differ from one run to the other. To minimize this effect and in order to select the best factorization results, it is crucial to repeat the process using different random initialization for matrices **W **and **H**. Standard NMF module provides this functionality using two methods: 1) repeat the process a predetermined number of times and select the best possible solution (the ones that maximizes the explained variance) 2) Combine different random runs in a single output file, as proposed in [18] (see Figure 2).

Standard NMF is therefore a wide-ranging analysis module that is not specifically focused to any particular analysis but more generally oriented to any potential application that might use this factorization method for analysis.

### Gene expression bicluster analysis

One of the main goals in the analysis of large and heterogeneous gene expression datasets is to identify groups of genes that are co-expressed in subsets of experimental conditions. The identification of these local structures plays a key role in understanding the biological events associated to different physiological states as well as to identify gene expression signatures. Classical one-way clustering techniques, especially hierarchical clustering, have been commonly applied to cluster genes and samples separately in order to identify these types of local patterns. In the last few years, many authors have proposed the use of two-way clustering methods (also known as biclustering algorithms) to identify gene-experiment relationships. For a review see [24].

bioNMF estimates biclusters using a novel method based on a modified variant of the Non-negative Matrix Factorization algorithm which produces a suitable decomposition as product of three matrices that are constrained to have non-negative elements. The new methodology, denoted as Non-smooth Non-negative Matrix Factorization (*ns*NMF) has been recently presented in [20], and its application in biclustering gene expression patterns has also been reported in [10]. *ns*NMF can also be interpreted as a parts-based representation of the data due to the fact that only additive, not subtractive, combinations of data items are allowed. In particular this matrix decomposition produces a sparse representation of the gene expression data matrix, making possible the simultaneous clustering of genes and conditions that are highly related in sub-portions of the data.

Similarly to NMF, the non-smooth non negative matrix factorization model is used to approximately reproduce a gene expression matrix **V **with *m *genes and *n *experimental conditions as a product of three new matrices **W**, **H **and **S **(**V = WSH**), with dimensions *m*×*k*, *k*×*n *and *k*×*k *respectively where *k *is the rank of the factorization. The *k *columns of **W **have the dimension of a single array (*m *genes) and each row of **H **has the dimension of a single gene (*n *experiments). Matrix **S**, on the other hand, is denoted as smoothing matrix and its task is to demand sparseness in both **W **and **H**.

For details of the algorithm see [10]. Due to the sparseness constraint imposed by the smoothing matrix **S**, each factor obtained by *ns*NMF contains a relatively small set of genes with non-zero coefficients that determine a local gene expression feature. These genes behave in a strongly correlated fashion in a sub-portion of the data and constitute a gene module. In the same way, coefficients in **H **are used to determine the set of experimental conditions highly associated to these modules. In other words, the set of genes and experimental conditions that show high values in the same basis experiment (i^th ^column of **W**) and its corresponding basis gene (i^th ^row of **H**), respectively, are highly related in only a sub-portion of the data and constitute a gene expression bicluster.

**Figure 3:**
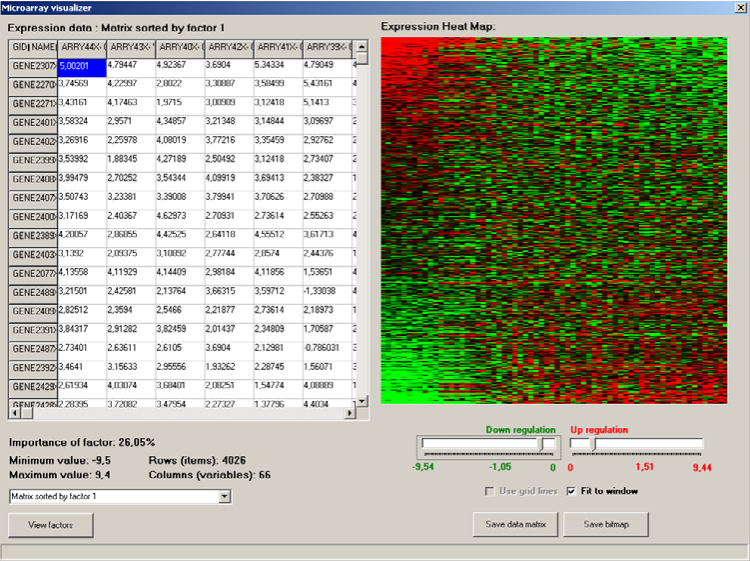
**Graphical User Interface for biclustering application**. Each factor is used to sort the original data matrix to emphasize the clustering structure of the data. Biclusters can be browsed in textual and graphical format. Thresholds to select the biclusters of interest can be interactively selected

Once the factorization has been completed, results can be explored using a graphical user interface (see Figure 3), allowing the selection of interesting patterns by removing those genes and samples with very low values in their factor and encoding vector. This is accomplished by sorting the original data matrix by basis genes and basis experiments, which creates a natural ordination in which genes and samples are arranged based on their association to a given local pattern. One of the advantages of the factorization model we are using is that its sparse nature reinforces those genes and experiments that significantly sustain the factor while masks those that do not add any value to it at the same time. Therefore, by simply applying a threshold on both genes and samples for a particular factor we obtain a bicluster. bioNMF offers the graphical interface for selecting biclusters using this criterion (see supplementary web site for details of the GUI).

Similarly to the standard NMF module, the biclustering process allows the multiple execution of the *ns*MNF algorithm with different random initialization conditions for matrices **W **and **H**. The solution that best reproduces the original data matrix is then selected for the analysis.

Regarding processing time, this algorithm takes one minute and twenty seconds in a 2.1 GHz Pentium M processor to process 1000 iterations with a data set containing 4585 genes with 46 experimental conditions.

### Sample classification

This module implements the approach proposed in [9] where NMF was used to classify tumors samples. This methodology uses NMF to reduce the dimension of expression data from thousands of genes to a set of metagenes to be used for identification of distinct molecular patterns and class discovery. In this particular application the NMF algorithm groups the samples into *k *clusters, being *k *the factorization rank.

To determine the most suitable number of meaningful clusters for a given dataset a model selection, that exploits the stochastic nature of the NMF algorithm, was also implemented in bioNMF as proposed in [9]. Since NMF might vary its results depending on the random initial conditions, it is important to evaluate the stability of the clustering structure of the data into *k *classes when running the algorithm a certain number of times using different random initialization.

This model selection method is based on the idea of consensus clustering [25], that quantitatively evaluates the fact that if a clustering into *k *classes is strong, it is expected that sample assignment to clusters would not vary significantly from run to run. The consensus matrix is estimated for a given factorization *k *by calculating the average connectivity matrix of a certain number of random runs. The connectivity matrix is a square *n*×*n *matrix, where *n *is the number of samples. Each entry *i*, *j *of this matrix takes the value of 1 if the sample *i *and sample *j *belong to the same cluster and 0 otherwise. This is determined in the NMF model by checking if both samples, *i *and *j*, which are represented by columns *i *and *j *of matrix **H**, contain a maximum value in the same factor (same row). The entries of the consensus matrix then range from 0 to 1 and reflect the probability that samples *i *and *j *cluster together. If a clustering is stable, it is expected that the consensus matrix will not to vary significantly among runs. Therefore, its dispersion between 0 and 1 measures the reproducibility of the class assignments with respect to random initial conditions.

**Figure 4:**
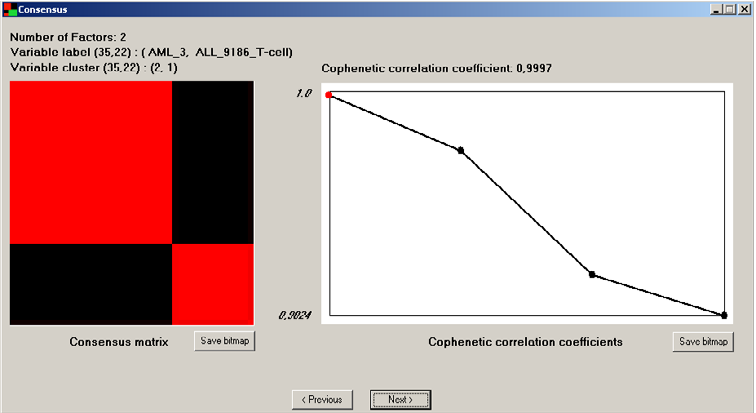
**Graphical User Interface for the sample classification module**. This panel shows the reordered consensus matrix and cophenetic correlation coefficient computed for each rank (*k*) used in the analysis. The figure shows the reordered consensus matrix obtained from 50 independent runs of NMF at *k *= 2 for the leukemia data used by Brunet *et al*. Graph on the right side represents cophenetic correlation coefficients obtained for *k *= 2-5.

The samples (rows and columns) of the consensus matrix are then reordered using the average linkage method to provide visual insights of the clustering stability (Figure 4). In addition, a quantitative measure of the matrix dispersion is also calculated based on the cophenetic correlation coefficient [9]. This coefficient equals 1 for a perfect consensus matrix (all entries 0 or 1) while tends to 0 when the level of dispersion of the consensus matrix increases. According to [9], the proper values of *k *should be selected where the magnitude of the cophenetic correlation coefficient begins to fall (Figure 4). This methodology provides a general method for robust molecular pattern discovery than have been effectively used in [9,11,15].

bioNMF fully implements this methodology using the divergence-based update equations [26] to solve the NMF problem, as was proposed by Brunet *et al*. [9]. Figure 4 shows the results of this method in classifying acute myelogenous leukemia (AML) and acute lymphoblastic leukemia (ALL) [27]. This data set has been used in [9] when this classification approach was reported.

It is important to mention that the cophenetic correlation coefficient has also been used to estimate the optimum factorization rank (value of *k*) in the biclustering application [10] and in the analysis of semantic profiles using the scientific literature [18]. This module can then be used as a previous step to for the rest of the analysis modules included in this application.

This methodology has an important computational efficiency drawback due to the fact that a large number of runs per factorization rank (*k*) are needed for the sake of robustness. Even thought bioNMF implements a fast version of this method (which takes less than 25 minutes for the analysis described in figure 4 in a 2.1 GHz Pentium M processor), careful attention should be paid when using this method with large datasets.

## Conclusion

Non-negative matrix factorization method has gained high popularity in the Bioinformatics field due to its potential in providing new insights about the complex relationships in large data sets. Although this algorithm is conceptually simple, its use by the scientific community still demands a certain level of programming skills to fully exploit it. The bioNMF application aimed at filling this gap by providing the research community with a tool containing the functionality needed to run either a simple exploratory analysis or to answer more complex analysis questions in an easy-to-use environment.

Current implementation of bioNMF includes a basic functionality for running the original NMF algorithm, which can be easily used with any data set. To demonstrate the usefulness of this method we described different types of analysis that have been proposed in different experimental contexts. This includes applications for finding functional gene modules [9,13], analysis of protein sequences [16] and extraction of semantic features from the scientific literature [18].

More concrete applications of NMF have also been included in bioNMF tool. For example gene expression biclustering, which has been incorporated in this application using a new sparse variant of NMF [10]. Additionally, sample classification using a robust classification methodology is also implemented [9]. Although some of the analysis modules implemented in bioNMF are based on complex methodologies, the whole analysis process remains very intuitive and simple for a final user.

There are still open problems; however, that requires a more detailed study. That is the case of the available methods for making the data non-negative, in particular, for the gene expression applications described in this work. In this application we have implemented four methods to cope with this problem. Nevertheless, we believe that there is no best method for all applications and results are very much dependent of the data and problem. A full comparison of methods to transform gene expression data into positive data sets is more than welcome and it represents an interesting topic of research.

bioNMF will also be systematically updated to support new functionalities and applications that might potentially help in the analysis of biological information using this methodology or some of its variants. In this way we expect that this tool helps researches in this field in using a method that it is conceptually simple and powerful for the process of data analysis.

## Availability and requirements

**Project home page**: 

**Source code availability**: 

**Operating system**: Microsoft Windows (98, Me, 2000, or XP)

**Programming language**: Delphi Pascal v.7

**Other requirements**: 1024 × 768 resolution

**License**: GPL

**Any restrictions to use by non-academics**: none

## Abbreviations

**NMF **– Non-negative Matrix Factorization

**PCA **– Principal Component Analysis

**SVD **– Singular Value Decomposition

**ICA **– Independent Component Analysis

***ns*NMF **– Non-Smooth Non-negative Matrix Factorization

**AML **– Acute Myelogenous Leukemia

**ALL **– Acute Lymphoblastic Leukemia

**GUI **– Graphical User Interface

**GO **– Gene Ontology

## Authors' contributions

APM, RDPM and PCS conceived the study. APM and RDPM designed and developed the software. PCS, JMC and MC developed the tests and documentation, FT developed the computational optimization of the method. APM, JMC and RDPM managed and coordinated the project. All authors participated in writing and revising the final manuscript.
